# Retrospect

**Published:** 1818-07

**Authors:** 


THE LONDON

### Medical and Physical Journal.

1 OF VOL. XL.] JULY, 1818. [NO. 233.

"For many fortunate discoveries in medicine, and for the detection of numerous errors, the world is indebted to the rapid circulation of Monthly "Journals; and there never existed any work to which the Faculty in "EUROPE and AMERICA were under deeper obligations than to the "Medical and Physical Journal of London, now forming a long, but an "invaluable, series."—RUSH.

### *Retrospect.*

IT is not easy for us to offer much in this department without paying an ill compliment to our own industry, or to the memory and attention of our readers. We shall, however, endeavour to fill up a few gaps which may have escaped us, and which cannot be so easily attended to, unless works of a similar description should come in our way.

Were we to detain our readers with all the physiological reveries or experiments at home and abroad, we might instruct them by proving the folly of attempting to show what never can be demonstrated, or, if known, never can be turned to any use. To what purpose are we to engage in the uncertain experiment of proving which part of an animal dies first. The connexion between the various parts, during life, is highly important, by the instruction we derive from sympathetic symptomalology. So far some advantage may be derived from experiments to prove in which part, or in which series or organs, action first ceases under the application of different kinds of violence, or of different kinds of poisons. From such experiments, well directed and explained intelligibly, as we are induced to expect may hereafter be the case, we may learn the kind of narcotic suited to different diseases, and to the different organs as they become diseased. Till this is accomplished, we feel ourselves unable to proceed as we could wish in appreciating the labours of some philosophers whose works have been long on our shelves. Dr. Philip, in his last volume, extols, indeed, the discoveries of Mr. Hunter; but it is in confirmation of his own experiments on digestion, most of which might have been omitted altogether, the result having been sufficiently proved. As, however, the conclusion of his volume contains many pathological remarks, we shall, in a future Number, confine ourselves principally to them.

In a pathological view, the two subjects which have principally occupied the attention of the medical public are, Nosology, and the cure of Syphilis without mercury. We are not afraid of being accused by our readers of inattention to either of these subjects. To the first, as connected with logical precision, we shall gladly pay further attention; but, as Mr. Good has not thought our reply worth his notice, we must leave the points in dispute to the impartiality of others.

We hope the controversy concerning the nature of pus, and its formation, will not be hastily abandoned. Lest our partiality to Mr. Hunter should seem to influence us, we shall admit of nothing but fair controversy; and, as in the case of Mr. Good, each party shall have the advantage of a month's possession of the mind of our readers.

The formation of pus being connected with absorption as the means of ulceration, there is a question in this branch of physiology which we are obliged to notice; and, in transcribing largely from a contemporary Journal, we hope at once to show our respect and impartiality. An ingenious writer in the Repository undertakes to prove, that the "absorbents, in the execution of their functions, are *perfectly passive*, and that the process of absorption is referable to some principle *independent of life*, *to some universal law of nature resulting from the construction of the parts concerned in the absorbent functions*." In proving the above position, the generally-received opinion is stated in the following words:

"The properties which are universally admitted as belonging to the absorbents are these:—First, that of being enabled by an *active power* of their own, a power, as it is termed, *inherent in their coats*, of conveying materials to the circulation; secondly, of varying, by incurring directly an increase or diminution in the energy of their functions; and, thirdly, they are supposed to possess that peculiar kind of sensibility by which they are enabled to absorb or to reject substances, as such substances may prove either beneficial or injurious."

Most modern physiologists admit, it is true, the two first propositions; but it is in effect only, and not in cause: for, whether this effect is produced by the coats of absorbent vessels, or by what means, cannot, nor is it necessary that it should, be ascertained. It is enough that experiment proves these vessels have an absorbent power. The third proposition has long since been exploded in all the modern schools, as inconsistent with facts of daily occurrence.

The writer proceeds to show that these vessels can have no contractility, as Dr. Parry has proved that the arteries have none. We have before given our opinion of the inconclusiveness of Dr. Parry's experiments in this respect; but we are ready to concede to our author the impossibility of proving a contractile power in the absorbent vessels, and that it is only presumed by implication,—nay more, we scruple not to shelter ourselves under *the indolent adoption of the phrase of the* "power of life," (excepting that we cannot admit that it is entirely an unmeaning phrase.) For, if we ascertain properties during life, and that these properties cease with life, we must consider that such properties as altogether the effect of life. At the same time, we conceive ourselves no way obliged to explain what life is, nor by what means it produces such effects, but only to trace those properties which distinguish animal matter, during life, from those which remain after death. Thus, to trace the life of one part of an artery, we examine what power it has of contracting beyond its elasticity; and, as we find this power gradually ceases with life, and that its elastic power continues as long as its texture remains to our senses unaltered, we impute the first power to life, and the latter to texture only. Very soon after the cessation of those actions by which life is supported, we find that no stimulus we can apply to a muscle will induce a contraction which was easily excited as long as life continued. Hence we conclude, that the contractile power of a muscle is a property of life. In answer to this, we have an instance given us in which the absorbents showed a greater degree of action after death than during life.

"If (says this gentleman) I shall be called upon to cite instances tending to the illustration of these remarks, I have to relate, that I have long since observed, and, I dare say, in common with many others of the profession, that in cases in which the patients have perished from ascites, the abdomen after death has suffered so considerable a diminution in size, as to warrant the conclusion, that a quantity of water, subsequently to the extinction of life, had been removed by the absorbents."

The author has observed the same, though with less certainty, in hydrothorax and in abscesses.

We are ready to acknowledge that these cases have not occurred to our observation: on the contrary, we have often found transudations of fluids into cavities where, during life, we had no reason to expect them. We are not, however, disposed to question the writer's veracity or accuracy; we would only remark, that *almost* in *articulo mortis* many diseased actions seem to cease, and healthy actions to be restored. In cases of obstinate constipation, it is not uncommon to find the peristaltic motion become regular as soon as respiration has ceased; and, in some instances of dropsy, the kidneys and absorbents have been found to recover their action as an immediate prelude to the death of the patient; but not, we acknowledge, in our observation after respiration has ceased. On this subject, however, we must submit to the fidelity of the author. Let us now, then, examine the necessary inferences from his own fact, and from his general position.

If the power of absorption is really passive, like elasticity, or inherent in the coats of absorbent vessels unconnected with life, it seems strange that there should be any difference in their action, and, consequently, that they should absorb so much more at one time than at another, and even better after death than during life. Though a cartilage retains its elasticity after death, we never find that power increased, or in any way altered, during life or after death. It must, however, be admitted, that, during life, the elastic force of cartilage is controlled by muscular force. We shall, therefore, if the author please, suppose that the absorbents are influenced in their actions, during *life*, by some other organs. Yet still this same *life* will come in our way. Let us then dismiss it altogether, and then we shall be obliged to inquire why, in all cases of death, the absorbents, being now left uncontroled, do not exert their full power, and why all effusions are not thus absorbed.

If ever we should find that the absorbent vessels have really this inherent power, it is not easy to calculate the advantages which may arise from their application to the arts. By the proper management of the lymphatics of the turtle, may we not expect water to be raised to any height without the assistance of pumps? What may we not accomplish with the thoracic duct of a horse, or of an elephant, or of the camelopardalus? But it is time that we should return to Mr. C. Bell's speculations on the venereal disease. It is but justice to admit that we were much pleased with his *exordium*.

"Just about the time when a safe rule of practice in the treatment of the venereal disease had been established among judicious surgeons—when the experiments and theories of Mr. Hunter had served to give a more correct idea of syphilis, and a simple mode of practice, certain opinions came into fashion, which have increased in extravagance from day to day, and have been attended with the very worst consequences. The question has all at once assumed unusual importance, in consequence of some gentlemen of authority in the army outstripping their brethren in the civil department, and having somewhat suddenly (on the ground-work of these dangerous opinions) commenced practice on that extended scale which the peculiarity of their situation puts within their power.

"It is impossible (continues the author,) to see so large and valuable a part of the community exposed to hazards, greater than the worst perils of the field, without feeling much anxiety."

In this last apprehension we feel less than Mr. Bell; because, as is afterwards admitted, the venereal disease being usually slow in its progress, the delay of a few weeks, in order to ascertain facts, appears to us more than venial in all cases of doubt, especially in subjects whose confinement from business is often unimportant. So far, however, the correctness of Mr. Hunter's experiments and theories is admitted to have introduced a safe mode of practice; and we were led to suppose, that it was only an attempt to carry these opinions to an extent beyond their meaning which produced all the mischief. Soon, however, the author proceeds to give us another proof* of his total ignorance of Mr. Hunter's doctrines, and of the facility with which he condemns what he does not take the trouble to understand.

"These doctrines (says Mr. Bell, in a note,) are not referred to any precise authority, but every one knows that they take their origin in certain opinions of Mr. Hunter, or from certain suggestions in his Treatise on the Venereal Disease. Looking to that work, and the overstrained eulogy of the author's opinions, it is impossible not to ascribe in some degree to this source those opinions and that practice which, I feel myself called upon to say, is dangerous. It is much to be lamented, that there is too often a disposition to admire, in the great men who have gone before us, some unsubstantial theory or opinion thrown out casually, rather than those solid and better parts of their professional exertions, for which the world stands indebted to them. I know that I am accused of wanting due deference for the opinions of Mr. Hunter. If it were so, it would be a lesser offence than theirs, who bestow upon him indiscriminate praise, concealing from the younger part of the profession how apt his expressions are to mislead them on practical subjects. In reference to the present subject, it cannot be denied, and ought not to be kept concealed, that, with all its merits, Mr. Hunter's Treatise on the Venereal Disease is no guide for practice; and yet, young men learning that it is a book justly esteemed, and that Mr. Hunter was the first surgeon of his day, they naturally think, that they have got in that volume a treasure of sound doctrine and safe practice."

As all this is particularly directed to Mr. Bell's pupils, or to the younger part of the profession, how desirable would it have been had he produced any specific practice or specific "doctrine that is unsound, or any practice, recommended

* See Mr. Bell's anatomical works *passim.*

by Mr. Hunter, that is unsafe!" We are ready to admit that Mr. H. told us of many diseases mistaken for the venereal, and some which would not yield to mercury, but he never called these venereal. No! his predecessors, who at various times attempted to cure the venereal disease by guaiacum, by sarsa, by opium; and his successors, who fancy they cure it by acids, and, last of all, without any remedies whatever,—it is they who are answerable for the consequences. Mr. Bell next adverts to some who conceive that "the venereal disease is never formidable, excepting when combined with the pernicious effects of mercury on the constitution, and even that the secondary symptoms appear only in cases where mercury has been given." If such opinions are entertained, we can only say, we have never discovered them in Mr. Hunter's writings, nor do we recollect the authors who entertain them.

The first of the following paragraphs contains much good sense, and we are only concerned that it is succeeded by three others, which conclude the paper, and leave us in the greatest terror, encreased by the most profound darkness.

"In conclusion, in an unlucky hour it has been said that the proof of a venereal sore is its not healing without mercury. On this unadvised expression, has there been built this new practice for the British army. On the other hand, I affirm, that with cautious intelligent surgeons, the *rule has always been to take care that you do not heal the sore by your applications, until you have ascertained its nature.*

"As to the other point, of our being able to remove a certain train of secondary symptoms without mercury, neither is this to be doubted. But no man can affirm what he has no means of ascertaining—that, after the prevalence of secondary symptoms for six months, he has, by such method, cured the patient of that disease. In some men, and especially in old men, the disease is of very slow progress; we see it break out in them very long after the infection. We see the disease propagated to the offspring after the recollection of the attack is lost, and would almost be tempted to fall into the opinion of those, who think that the adult constitution may sometimes oppose the influence of the disease, while it is exercised on the fœtus.

"Of all the statements of Mr. Hunter, none is more important in practice than this,—that we can destroy the active state of the disease, and yet not the latent disease. Mercury cures secondary symptoms, and yet we cannot absolutely, and to the last degree of certainty, say, that even with mercury we can *cure a pox thoroughly rooted in the constitution.*

"Now the disease is active when it is entering the constitution, when the primary symptoms are about to become secondary symptoms. Then by mercury we can assure the patient's safety; afterwards it is a strong probability that you shall, but it is no longer a certainty. It is this consideration, more than any other, which makes me desirous that my pupils should have a correct notion of the treatment of a chancre."

Mr. Bell may have seen what Mr. Hunter, who devoted a much longer life to the closest observation, never discovered, namely, "the disease propagated to the offspring after all recollection of the attack is lost." Whoever reads this, and the few lines following, if he believes the doctrines they contain, must be consigned to a life of perpetual horror: for all which, in our opinion, Mr. Bell is chargeable. Nor is this all: in the concluding paragraph, Mr. Hunter's doctrine, founded on the experience of more than thirty years, is rendered obscure by the introduction of the undefined terms of "active and latent;" and, last of all, the whole is placed beyond all the rules of sound reasoning, by the application of the laws and language of a science different from pathology.—What is meant by a pox thoroughly *rooted* in the constitution? Does the author intend the succeeding paragraph as an explanation? If so, we advise him not to suppose that the youths around him comprehend his meaning because they are silent. As *they* may be respectfully mute, we shall remind him, that the *roots* of a disease and its *eradication*, mentioned in his preceding page, are words to us incomprehensible, and make us suspect that he has never arranged his own ideas. In Mr. B.'s next number, we hope to find Mr. Hunter understood, and the subject treated logically. We conclude with offering this last hint to others, who may engage in a subject only complicated for want of the above requisites.

After these general remarks on subjects connected with our own labours, we shall attend to what has been done by others. In the anatomical department, M. Delondre gives a case in which there was a perforation in the septum ventriculorum of the heart of an adult.* The subject, a wife about nineteen years old, had experienced frequent syncope from the age of puberty, with palpitations and other formidable symptoms.

"That the aperture of communication, existing in this case, between the ventricles was really a congenital peculiarity, and not a lesion of structure consequent on diseased action, an attentive review of the slight history of it, which our limits only allow us to trace, will, we apprehend, suffice to convince the reader. The subject of the case alluded to was a lady, aged nineteen, who had experienced *from her infancy* habitual palpitations of the heart, aggravated by every kind of physical effort and moral affection. From the period of puberty, she sustained attacks of syncope, with vehement palpitation and other formidable symptoms, by which abortion was twice induced, and which progressively increased in violence to the last. The state of the pulse is but once distinctly mentioned: it was then 150, and scarcely perceptible; respiration tumultuous. The ascites and œdema of the limbs, supervening at an advanced stage of the disease, were completely, though not permanently, removed by frictions, with tincture of digitalis employed on the abdomen and inside of the thighs. On dissection, the pleura contained four pints, the pericardium about one pint, and the peritonæum (which, in some places, was of a red colour), from six to eight pints of serum. The lungs, particularly the left, were gorged with thick black blood, and had acquired the consistence of liver. The substance of the heart, which exhibited thrice its natural volume, was very flaccid, and readily lacerable. Its right ventricle was extremely thin and much dilated; the left thickened and contracted: and in the centre of the septum, which divides them, was seen an orifice of communication, measuring at least one inch, of an elliptic figure, and furnished in its whole circumference with bodies of a fibrous structure."

---

* *Recueil Periodique de la Société de Medecine à Paris. Avril* 1817.

If this was an original malformation, and produced so much misery, it seems difficult to conceive how the patient existed so long. We could wish that the description had been attended with a more accurate account of the other parts of the source of circulation. We have never met with this communication between the ventricles at an advanced age, and attended with palpitations without other malformations, and have suspected that the communication has been kept open on account of other difficulties in the circulation.

We should not have expected that so grave and learned a body as the French Academy should have engaged in so futile a controversy as that of the secretion of milk from chyle, without going through the process of sanguification, especially when founded on the absurd notions of Richerand. M. Salion has, however, had the patience, or industry, to answer all the arguments of M. M. Richerand and Gerard. We can hardly say more than that they rest their arguments on finding substances in the milk which cannot be detected in the blood, on the certainty that substances are conveyed, but little altered in smell, from the stomach to the mammæ, and on the rapidity with which the mammæ swell after the stomach has been filled even with fluids. Is it worth while to answer, that most of the secretions contain substances which the blood does not discover; that the perspiration often smells of substances taken into the stomach; and that, after the latter is empty, the reception of any solid or liquid instantly restores, rouses, or improves, every vital process?

The French still continue their experiments on vegetables. We are not altogether satisfied with them till they have been repeated, nor are the results of any considerable pharmaceutic importance. We have already mentioned their experiments on Ipecacuanha. Those on Opium have attracted the attention of Orfila and Magendie. Gautier has been engaged in Pyrethrum. We may hereafter say more on these subjects.

It has been anciently remarked, that there is nothing new under the sun.—When we find our neighbours engaged in experiments on distilled sea-water, and anxious to prove that it is by no means unwholesome, we cannot help reminding our countrymen of experiments repeatedly made on this side the channel. That fresh water thus procured is not unwholesome, has been long since ascertained; and the facility of procuring it, by the distillation of all the water used for culinary purposes on board ship, is equally well known: but still great objections remain. The salted provisions so boiled are rendered still more salt, or, at least, part with very little of the muriate of soda, which renders them less digestible, less palatable, and consequently less nutritious: Besides, the water is by no means free from other impregnations which are unpleasant to the taste, though probably not injurious to health.*

A more important article by Humboldt, in the same collection, is the account of the tree called *Palo de vaca*, from its property of secreting milk. Among the vast number of curiosities with which that valuable writer has enriched the world, those which may be subservient to food claim the first attention. This paper abounds with so many useful hints, that we shall hereafter transcribe it into our Collectanea.

We shall conclude this short Retrospect with remarking, that our continental neighbours talk less of *adynamic fevers,* (a term on the whole less objectionable than our typhus,) and describe their fevers as chiefly inflammatory, ataxique, or dysenteric. In the Transactions of the Dublin College, we meet a vast mine of useful knowledge concerning fever, the question concerning its contagious properties, and the mode of prevention. Nothing but a great press of matter has prevented an earlier notice of these valuable papers. Though this volume has attracted less attention than the Hospital Reports, from the same meridian, it contains, in our opinion, matter beyond all comparison better digested, and infinitely more applicable to the principal object of all medicine. The progress of the epidemics is well pointed out, and the means of prevention marked with that judgment

* "*Annales de Chemie.*"—[We had collected for our Retrospect an epitome of the discoveries in chemistry for the last eighteen months; but the great number of original articles oblige us to defer it till the next Number.—Edit.]

which evinces a thorough knowledge of, and close attention to, causes and effects. Dr. O'Brien, with a manly dignity, urges the absurdity of expecting any advantages from their Fever Hospital, beyond that of removing a piteous, not to say disgusting, object from assailing us in the most conspicuous parts of the public streets. At the commencement of his paper, he observes that the admissions have annually increased since the foundation of the hospital. Such a hint as this he considers sufficient, as introductory to his subject. Towards the close, he very justly remarks, that the sources of the disease are not any specific contagion like the exanthemata, but will be found in the habits and condition of our neglected sister kingdom. Until some changes are effected in the morals and habits of the labouring class, the cause will always exist, and its effects be proportionate to that cause. In times of greater distress it will be greatest, and will seem to disappear from its mildness in genial seasons, attended with plenty and facility of procuring employment. At more unfavourable seasons, a hospital becomes a depot, not merely of patients, but of infection; and, not only a depot or reservoir, but a means of increase. We have seen, in the accumulated misery from the Walcheren expedition, how many medical men, and others attached to the hospital, became victims to the concentrated fomes issuing from so much disease. In the Dublin Hospital, we are told that an apothecary and a physician, with eleven others attached to the house, were affected with the fever, and some to an alarming degree. In the Fever-house of London, we have witnessed the death of Dr. Murray soon after its establishment; since that, of several officers, and, lately, of that promising young physician, Dr. Da Costa.

